# Obstetrics

**Published:** 1909-12

**Authors:** D. C. Rayner


### OBSTETRICS.

**The occurrence of red degeneration in fibromyomata of the uterus during pregnancy.**—Considerable attention has been recently directed to the occurrence of this remarkable change in uterine fibroids. Although the phenomenon is met with in fibroids of the non-gravid uterus, its occurrence is more frequent, and the characteristic signs more marked, when associated with pregnancy. Two features which stand out prominently are pain and tenderness. One remarkable feature about the pain is the suddenness of its onset; indeed, it is sometimes so severe and its onset so sudden that it resembles the pain experienced by patients when an ovarian tumour twists its pedicle, or a gravid Fallopian tube bursts. Abdominal section has been performed quite a number of times under the supposition that one of these conditions was present, when at the operation it has been found that all the symptoms were due to red degeneration occurring in a uterine fibroid. Another interesting point with regard to the pain is that it is only present during the early stages of the degenerative process, that when the tumour is completely disintegrated the pain ceases. In a uterus containing multiple fibroids it commonly happens that only one of them exhibits the red change, and becomes painful, while the other remains colourless and insensible. If a fibroid undergoing this change be examined, it will be found that instead of being of a pale yellow or white colour it has assumed a deep red or mahogany tint. In the early stages the change in colour is exhibited in streaks, but as the degeneration advances the whole tumour becomes affected, and in well-marked examples becomes quite soft and diffluent. An interesting point is that no micro-organisms have been found, and this is the more surprising when it is remembered that many of these tumours have a somewhat offensive odour. On microscopic examination the tissue of the fibroid is found to be necrotic and does not stain; the characteristic redness is due to the diffusion of blood pigment through the necrosed tissues. The softening of the tissue composing the tumour sometimes takes place with great rapidity, so that even the hardest fibroid becomes in a very short time quite soft and diffluent. With regard to treatment, it is pointed out by Bland-Sutton,[1] in a very instructive paper on this subject, that when pain and tenderness are present in a mild degree they generally subside if the patient is kept at rest in bed. If, however, the symptoms are severe prompt surgical treatment will probably be necessary.

The mortality from **puerperal sepsis** in England is still high. It is estimated that in England and Wales at the present time more than 2,000 women lose their lives every year from this cause. At the recent meeting of the British Medical Association Dr. Thomas Wilson[2] opened an important discussion on this subject, and reviewed the various modern methods of treatment. The most severe form of puerperal sepsis usually arises during the first few days after delivery, and at the onset it is very difficult to say whether we are at the commencement of a serious or even fatal illness or merely a passing toxæmia. The rise of temperature

[1] *Brit. M. J.*, 1909, i, 1471. [2] *Ibid.*, ii, 1037.

is usually the earliest and most convenient symptom on which to decide upon treatment. In cases where the temperature rises to 102° F., and a careful examination fails to discover any obvious cause, such as an inflamed breast, sore throat, etc., it is recommended that an intra-uterine douche should be given. Of the various solutions used he prefers iodine, one drachm to the pint, five or six pints being used. If the temperature does not drop within twelve hours further treatment should be undertaken. The indication is to make certain that no putrescible materials, such as clot or portions of placenta or membrane, are retained in the uterus. In attempting to fulfil this indication we find that practitioners are fairly sharply divided into two classes, those who advocate the use of the curette and those who rely upon digital evacuation. The objections to the curette are that when applied to the interior of the puerperal uterus new lymph and blood channels are opened up which offer the germs easy access to the deeper layers of the uterine wall, and also by its use the protective barrier of leucocytes, which form the first line of defence against bacterial invasion, is removed. Taking everything into consideration, digital evacuation presents solid advantages over the use of the curette. When local measures, douching and digital exploration have failed, and the septic germs have penetrated more or less deeply into the tissues, the problem of treatment becomes much more difficult of solution. The indications are still to attempt to destroy the germs and their poisons, and to encourage and reinforce the natural powers of resistance of the body. Direct attempts to paralyse or kill the germs have been made by the injection into the circulation of such substances as formalin, corrosive sublimate or collargol. The obvious difficulty is to find a substance that shall be effective on the germs and still not injure the patient's tissues, and so far no satisfactory substance has been discovered. Indirect means of attacking the invading organism include attempts to favour the production and activity of phagocytes, which originate chiefly in the bone marrow, and which appear to play an important part in the defence of the body against bacterial invasion. The introduction of a large quantity of water or normal saline solution has been supposed to act in this way, and at the same time dilutes the poison and favours its excretion. Encouraged by the success that has followed its use in diphtheria, specific immune serums derived from animals have been used, but so far the results have been disappointing. More recently vaccines have been prepared from the invading organism actually present in the patient, and these have sometimes appeared to give good results.

Drs. Hartwell, Streeter and Green[1] record ninety-seven cases of sepsis treated with bacterial vaccines, eighteen of which were cases of puerperal infection, and all of the eighteen recovered.

[1] *Surg., Gynec. and Obst.*, 1909, ix, 271.

In four blood cultures were made. One showed streptococcus pyogenes, one staphylococcus pyogenes aureus, and two were negative. Fifteen showed streptococcus from the cervix in pure or nearly pure culture. In all the cases local treatment such as douching, curetting, etc., was undertaken, so that the exact value of the vaccine is somewhat obscured. In many of the cases, however, no improvement took place after the local treatment, whereas the change for the better after the vaccine was very marked. In every case the pathogenic organism was determined by culture, and in the majority an autogenous vaccine was used. The vaccines were made up according to the method devised by Sir A. E. Wright. The dose given varied from five to twenty-five millions. Occasionally as the dosage was increased reactions were seen; they consisted usually of slight headache and backache, accompanied in the more severe cases by nausea and vomiting, and a rise in temperature of a degree or two. They followed several hours after inoculation and disappeared again in a few hours. These reactions were rare, and as far as is known were followed by no ill-effects. Whenever they occurred the amount of the inoculation was reduced so as to avoid their repetition. Local soreness at the site of inoculation was frequently noted. In most of the cases the improvement after injection was most striking. The writers concluded, as a result of their experience, that bacterial vaccines should be employed in all cases of puerperal infection which do not immediately respond to routine treatment.

The success that has attended surgical intervention in such affections as peritonitis from disease of the vermiform appendix has encouraged a more extended trial of various operations in puerperal infection. The surgical measures that can be undertaken in suitable cases consist in incision and drainage when large collections of turbid sero-fibrinous or purulent exudations are present in the peritoneum. The best method of operating is still unsettled. Some surgeons open the abdomen either by one large or several small incisions and secure free drainage, while others advocate early and free opening of the peritoneal cavity by the posterior vaginal fornix. It is not advisable as a rule to remove infected uterine appendages in recent cases of puerperal infection, but to be content with simply opening up any purulent foci that may be present, and providing adequate drainage, reserving removal to some later date if it should be necessary. The operation of hysterectomy which has been advocated by some in cases of puerperal sepsis has proved to be very fatal, and is of course quite useless in cases of general infection. When infection has spread into the uterine or ovarian veins, giving rise to thrombo-phlebitis, the attempt has been made to cut short the disease by ligature or excision of the diseased vessels; the results, however, have been far from encouraging. The curative treatment of grave cases of puerperal infection is not, and from the nature of the disease probably never will be, satisfactory. Hope for the future must still centre round prevention.

D. C. RAYNER.

---

### Reviews of Books.

